# Estimating cancer risks due to whole lungs low dose radiotherapy with different techniques for treating COVID-19 pneumonia

**DOI:** 10.1186/s13014-021-01971-7

**Published:** 2022-01-20

**Authors:** Amin Banaei, Bijan Hashemi, Mohsen Bakhshandeh

**Affiliations:** 1 Department of Medical Physics, Faculty of Medical Sciences, Tarbiat Modares University, Al-Ahmad and Chamran Cross, 1411713116 Tehran, Iran; 2 Department of Radiology Technology, Faculty of Allied Medical Sciences, Shahid Beheshti University of Medical Sciences, Tehran, Iran

**Keywords:** COVID-19, Low dose radiation therapy, Cancer risk, Intensity modulated radiotherapy, 3D-conformal radiotherapy

## Abstract

**Background:**

Low dose radiotherapy (LDRT) of whole lungs with photon beams is a novel method for treating COVID-19 pneumonia. This study aimed to estimate cancer risks induced by lung LDRT for different radiotherapy delivery techniques.

**Method:**

Four different radiotherapy techniques, including 3D-conformal with anterior and posterior fields (3D-CRT AP–PA), 3D-conformal with 8 coplanar fields (3D-CRT 8 fields), eight fields intensity-modulated radiotherapy (IMRT), and volumetric modulated arc therapy using 2 full arcs (VMAT) were planned on the CT images of 32 COVID-19 patients with the prescribed dose of 1 Gy to the lungs. Organ average and maximum doses, and PTV dose distribution indexes were compared between different techniques. The radiation-induced cancer incidence and cancer-specific mortality, and cardiac heart disease risks were estimated for the assessed techniques.

**Results:**

In IMRT and VMAT techniques, heart (mean and max), breast (mean, and max), and stomach (mean) doses and also maximum dose in the body were significantly lower than the 3D-CRT techniques. The calculated conformity indexes were similar in all the techniques. However, the homogeneity indexes were lower (i.e., better) in intensity-modulated techniques (*P* < 0.03) with no significant differences between IMRT and VMAT plans. Lung cancer incident risks for all the delivery techniques were similar (*P* > 0.4). Cancer incidence and mortality risks for organs located closer to lungs like breast and stomach were higher in 3D-CRT techniques than IMRT or VMAT techniques (excess solid tumor cancer incidence risks for a 30 years man: 1.94 ± 0.22% Vs. 1.68 ± 0.17%; and women: 6.66 ± 0.81% Vs. 4.60 ± 0.43%: cancer mortality risks for 30 years men: 1.63 ± 0.19% Vs. 1.45 ± 0.15%; and women: 3.63 ± 0.44% Vs. 2.94 ± 0.23%).

**Conclusion:**

All the radiotherapy techniques had low cancer risks. However, the overall estimated risks induced by IMRT and VMAT radiotherapy techniques were lower than the 3D-CRT techniques and can be used clinically in younger patients or patients having greater concerns about radiation induced cancers.

## Background

Application of low dose radiotherapy (lower than 0.5 Gy) with photon beams to treat viral and bacterial pneumonia has a long history [1–3]. Recent studies suggested that similar approaches could be used for treating patients suffering from COVID-19 pneumonia [4–6]. There are also more than 10 registered clinical trials assessing this issue [7–16]; however, higher prescribed radiation doses up to 1.5 Gy were used in some of these studies [7, 9–13, 15–17]. Whole lung radiotherapy with external photon beams at low doses (0.5–1.5 Gy) is a novel treatment option, and the effectiveness of this method is under investigation in clinical trials. At the time of writing this article, two of the clinical trials reported that whole lung low dose radiotherapy (LDRT) could improve the health situation of the COVID-19 patients having severe pneumonia [18]. In contrast, one study reported that whole lung LDRT failed to improve clinical outcomes in critically ill patients requiring mechanical ventilation [19].

The main mechanism proposed for lung radiotherapy was the anti-inflammatory effects of low dose radiations [20]. Patients with serious COVID-19 pneumonia usually acquire acute respiratory distress syndrome (ARDS) and sequential organ failure. It was proposed that the main contributor for occurring ARDS, is “Cytokine storm” which is a high inflammatory response. This response is characterized by increased interleukins IL-2 and IL-7, and macrophage inflammatory protein (1-α). Details of this mechanism that invoke a cytokine storm were described by Mehta et al. [21]. Several studies suggested low dose radiotherapy can result in inhibition of inflammatory response. However, the significant inhibition effect on cytokine storm has not been proven yet [20].

Although the whole lung LDRT may be beneficial for COVID-19 patients, the radiation side effects, mainly radiation induced cancers, must be evaluated for different radiotherapy techniques. There are not any acute radiation effects for lung or other organs in the chest region at less than 7 Gy, and lung LDRT doses (0.5–1.5 Gy) are well below this threshold. Therefore, the radiation induced cancer risks must be estimated for lungs LDRT of COVID-19 patients suffering from pneumonia.

The goal of whole lung LDRT is to deliver a homogeneous dose to both lung volumes while sparing normal peripheral tissues and organs like heart, esophagus, and breasts. However, there is no study reporting normal tissue dose constraints at the dose levels of lung LDRT. There are several external radiotherapy delivery techniques like 3D-conformal, intensity-modulated radiotherapy (IMRT) with fixed gantry angles, and volumetric modulated arc therapy (VMAT) for irradiating lungs.

There are different models for estimating the cancer risks induced by low dose radiations [22–25]. BEIR VII-Phase2 (Biologic Effects of Ionizing Radiation) report presented a full review of the available biological, biophysical, and epidemiological investigations. It proposed the most up-to-date and comprehensive risk estimation for cancer induction from exposure to low-level ionizing radiations. The attributable risks of cancer incidence and cancer mortality for various cancer sites at different exposure ages could be calculated based on the preferred model proposed by this report [22]. This model estimated the risks for various organs regarding the sex and age of exposure. Therefore, we used this model for evaluating the cancer risks induced by lung LDRT of COVID-19 pneumonia patients.

Regarding our literature review, there is no study evaluating or comparing the dosimetric or radiation induced cancer risks at different radiotherapy protocols for whole lung radiotherapy of COVID-19 patients; therefore, in this study, the cancer risks induced by lung LDRT were estimated using the BEIR VII-phase2 preferred model for different radiotherapy delivery techniques.

An increase in cardiac heart diseases is another side effect of low dose exposures. It was shown that an increase of 1 Gy in cardiac mean dose resulted in a 7.4% increment in excess relative risk (ERR) of radiation induced cardiac heart diseases [26]. Therefore, the ERRs of these diseases caused by LDRT were also estimated for different radiotherapy techniques in this study.

## Methods

This retrospective study was performed following the relevant ethical guidelines and regulations, and the national ethics committee has approved the methods of this study. The computed tomography (CT) images of 32 patients (16 women and 16 men) with the mean age of 54.3 years and ranged between 32 and 74 years were used in this study without any intervention in the diagnostic or treatment procedures. All the patients whose CT images were used in this study had COVID-19 diagnoses based on clinical manifestations with a positive polymerase chain reaction of the nasopharyngeal swab, antibody test, and CT image manifestations.

The exclusion criteria were patients having a history of malignancy or heart failure and a history of radiation therapy or surgery in chest region.

### Planning

For each patient, four different delivery techniques were simulated, including 3D-CRT with anterior and posterior fields (AP–PA), 3D-CRT with eight coplanar fields, IMRT with eight fields, and VMAT with two full arcs techniques on the patients’ chest CT scans. The lungs, heart, breasts (for women), liver, stomach, esophagus, thyroid, spinal cord, and whole body were contoured on the patients’ CT images in the RayStation 8.A treatment planning software (RaySearch Laboratories, Stockholm, Sweden) under the supervision of an experienced radiologist. Dose distributions were calculated using the collapsed cone convolution algorithm in this software with 2 × 2 × 2 mm^3^ dose grids. The CT image matrix consisted of 512 * 512 pixels with a 2 mm slice thickness.

It must be mentioned that the RT-smartCTQA (dose.point GmbH, Germany) phantom was used to find the relationship between the Hounsfield Unit (HU) obtained from the CT system (Siemens 16-slice Emotion, Siemens Healthcare GmbH, Germany) and mass/electron density of different materials.

**Fig. 1:**
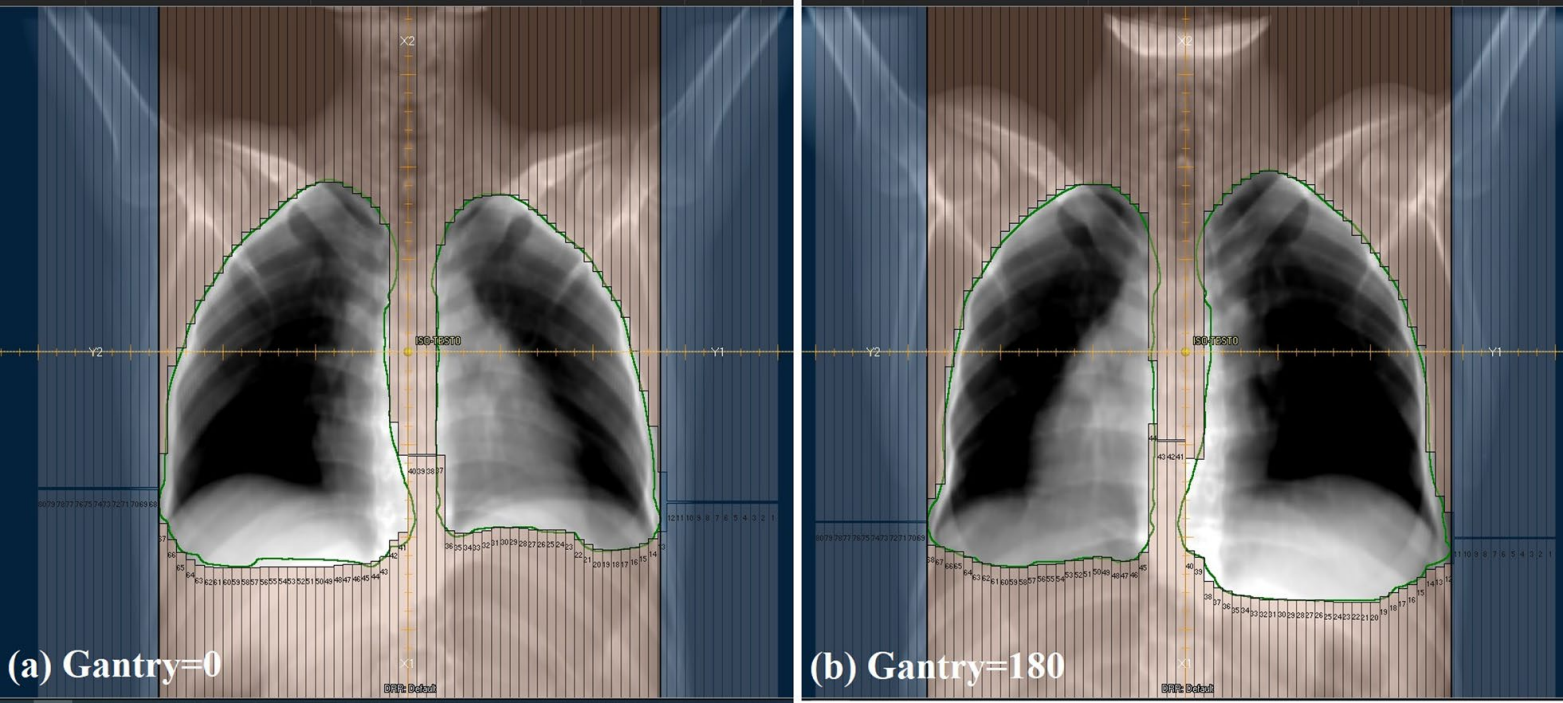
The beam eye views of treatment fields in 3D-CRT AP–PA technique using 160 leaves of MLC covering the PTV (specified by the green line) and sparing organs at risks located near lungs. The collimator rotated 90° for better sparing of peripheral structures. **a** Beam eye view of the anterior to posterior (AP) field (gantry angle = 0). **b** Beam eye view of the posterior to anterior (PA) field (gantry angle = 180)

In the 3D-CRT technique with AP–PA fields, two opposite 6 MV photon fields (anterior–posterior and posterior-anterior fields) at the gantry angles of 0 and 180 degrees obtained from Siemens Artiste linear accelerator (Siemens Healthcare GmbH, Germany) were used for irradiating at least 95% of PTV with 95% of the prescribed dose. Anterior and posterior fields’ weights were optimized to minimize hot spots. Furthermore, collimators were rotated to 90 degrees for better shielding of normal tissues around the lungs. The planning target volume (PTV) included both lungs plus a margin of 0.5 cm in all directions to account for setup and motion errors. Many studies reported the lung displacement in different directions, from 2.5 mm in anterior–posterior up to 18.5 mm in superior-inferior directions [25, 27–30]. Although lung volume typically changes by 10–25% [28], we considered a 0.5 cm margin for PTV because our assumption was that the motions in different directions for each patient could be estimated using 4D-CT and breathing gated imaging to have relatively small PTV margins. No additional dose constraints were imposed on any of the organs at risk. Figure 1 shows the beam eye views of treatment fields in 3D-CRT AP–PA technique. A multi-leaf collimator (MLC) covered the PTV while resulted in sparing organs at risks near lungs like the esophagus and spinal cord.

We used eight coplanar fields with the gantry angles of 0°, 60°, 90°, 120°, 180°, 240°, 270°, and 300° in the 3D-CRT technique with eight fields. The collimator angle and PTV margins were similar to the 3D-CRT AP–PA technique.

Eight fields IMRT technique with 6 MV coplanar photon beams at the gantry angles of 0°, 60°, 90°, 120°, 180°, 240°, 270°, and 300° was also used to irradiate at least 95% of the PTV with 95% of the prescribed dose. Leaf sequences using the step and shoot technique produced by Siemens 160MLC multi-leaf collimator (Siemens Healthcare, Germany) were generated for IMRT plans.

Two full coplanar arcs (180–179 clockwise; and 180–181 counter-clockwise arcs) with 6MV photon beams were used to generate VMAT plans with Siemens Artiste linear accelerator using Siemens 160MLC multi-leaf collimator (Siemens healthcare, Germany).

Inverse planning for IMRT and VMAT treatment plans, including defining the plan objectives, iterations, and final dosimetry (with collapse cone convolution algorithm) were performed in RayStation 8.A treatment planning software (RaySearch Laboratories, Stockholm, Sweden). The plan objectives for IMRT and VMAT plans were similar and were presented in Table 1 for PTV and other normal structures. It must be noted that there is not any dose constraint in the literature for lung LDRT; therefore the constraints used for optimizing IMRT and VMAT plans were selected to spare lung peripheral radiosensitive organs and cover the PTV with the prescribed dose.

**Table 1:** Dosimetric objectives used for IMRT and VMAT plans optimization for PTV and healthy structures

Organ	Objective	IMRT/VMAT
PTV (lungs + 5 mm margins)	V105%	< 5%
	V100%	≥ 95%
	V95%	≥ 99%
Heart	V90%	< 30%
	V60%	< 40%
	V20%	< 90%
Breast	V80%	< 5%
	V30%	< 20%
Esophagus	V90%	< 10%
	V50%	< 50%
Spinal cord	Max dose	< 95% of prescribed dose
Body	V105%	< 3 cc

Vx%: The percentage volume of the organ received at least x% of the prescribed dose

We evaluated 1 Gy to whole lung as a prescribed dose, however different dose prescriptions were proposed or COVID-19 Whole lung LDRT including; 0.5 Gy (in 1 fraction) [9, 17], 0.7 Gy (in 1 fraction) (10), 1 Gy (in 1 or 2 fractions) [7, 9, 11, 13], and 1.5 Gy (in 1 fraction) [18] based on current clinical trials and reports.

### Organ dose calculations and cancer risks estimation

Dose distributions and dosimetric parameters including homogeneity index (HI), conformity index (CI), mean organ dose, maximum dose of organs, and integral dose were compared between different delivery techniques.

For each patient, the organ doses were calculated after obtaining the dose distribution for each plan. The mean dose of the organs was used for cancer risk estimation based on the BEIR-VII report preferred model. This model proposed a linear no-threshold relation as the most reasonable description of the relationship between ionizing radiation exposure at low doses and the lifetime radiation induced cancer risks (up to age 90). This report considers moderating factors for cancer type, gender, age at exposure, and time elapsed after exposure [22]. A threshold-free linear model was used to estimate solid tumors, and a linear-quadratic model was used to estimate the risk of leukemia. The report uses an exponential multiple-risk estimation model of the natural risk frequency in the community. A combination of progressive and incremental models has been used to estimate the cancer risk based on age at radiation time (between progressive and incremental models). In some cancers, such as thyroid, the progressive model was applied. In some other cancers, such as breast cancer in women, the incremental model and the weighted mean of both methods were used to estimate cancer risk. In the expression of risk, the committee has finally presented the life attributed risks (LARs) [22]. These values were presented for cancer incidence and lifetime attributable risk of cancer mortality for the various sites of cancers at different exposure ages. The LAR is the difference in the rate of cancer risks between the exposed population and an unexposed population. It is an estimate of the probability of developing premature cancer from radiation exposure over the life of the subject. Thus, it depends on the subject’s age at the time of exposure and incorporates several additional factors such as the latency period from exposure to the first risk of cancer and the dose and dose rate effectiveness factor. These values present the additional risk of different cancers and the total risk of all cancers for ages (at the time of exposure) ranging from 0 to 80 years in both sexes for a dose of 0.1 Gy per 100,000 individuals.

There are several risk models developed for cancer incidence and mortality estimation, including ICRP [25], BEIR [22], United Nations Scientific Committee on the Effects of Atomic Radiation (UNSCEAR) [31]. We have chosen the BEIR VII model as it provides the parameters for specific organs for each sex and includes a parameter describing incidence with age at exposure and attained age. We evaluated the cancer incidence and mortality risks for adults (> 20 years old) because all the CT images used in this study belonged to adults patients.

Based on a previous study reported by van Derby et al. [32], the risk of coronary heart diseases increased linearly with increasing mean heart dose with a mean excess relative risk (ERR) of 7.4% per Gray (20.0% /Gy for ages < 27.5 years; 8.8%/Gy for ages of 27.5–36.4 years; 4.2%/Gy for ages of 36.5–50.9 years). Regarding this model, the excessive risks of radiation induced coronary heart diseases were calculated for different radiotherapy techniques of lung LDRT.

### Statistical analysis

Dosimetric parameters, including mean and maximum organ doses as well as HI and CI for PTV dose distribution [33], and estimated radiation induced cancer and coronary heart disease risks for different delivery techniques (i.e. 3D-CRT AP–PA, 3D-CRT 8 fields, IMRT, and VMAT) were compared using the repeated measurements and paired t-test statistical analysis.

The level of statistical significance was set at *P* < 0.05, and all the statistical tests were performed in SPSS (Statistical Package for the Social Sciences) software package, V18 (SPSS Inc., Chicago, USA).

## Results

**Fig. 2:**
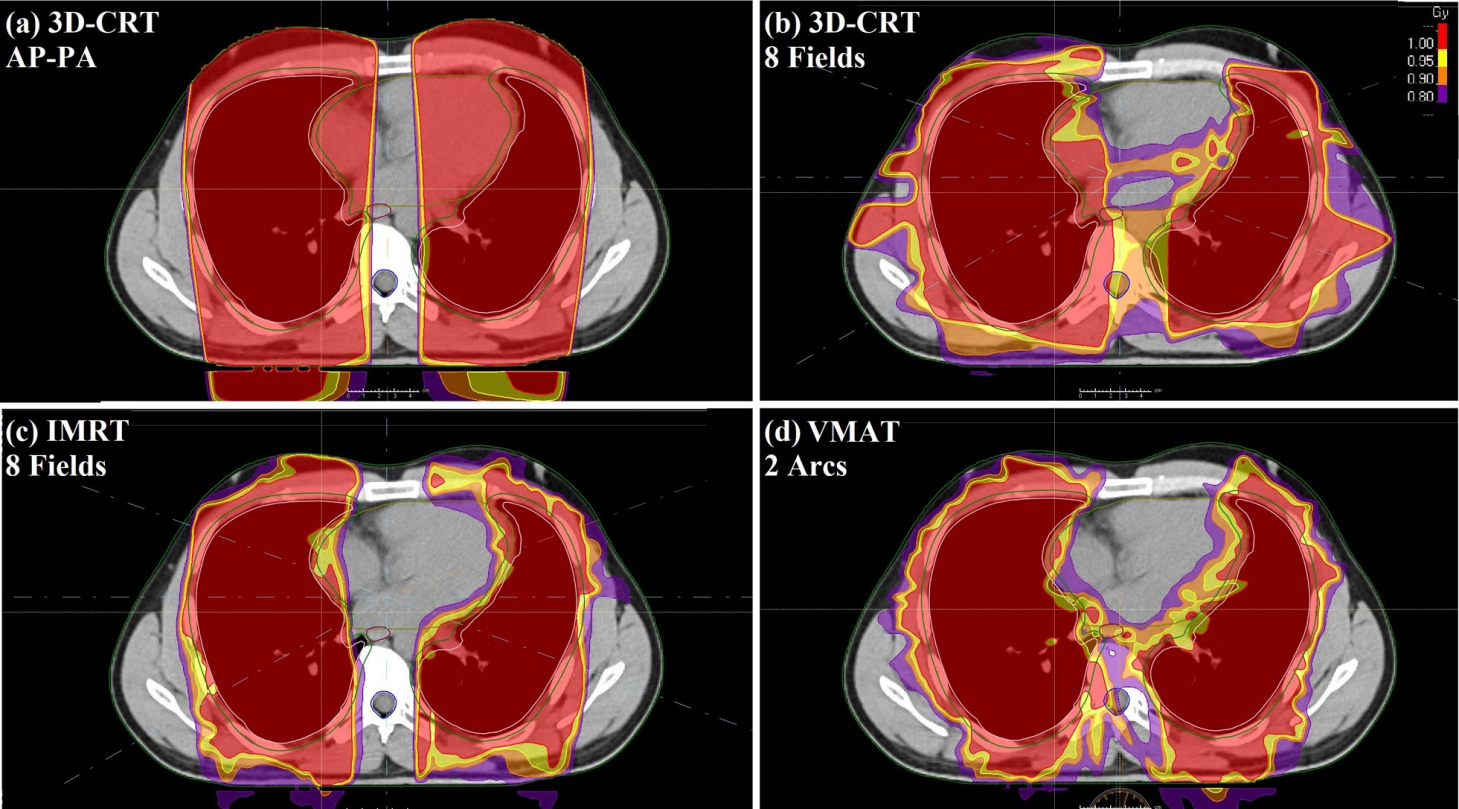
Dose distribution of whole lung LDRT with different delivery techniques and 1 Gy prescription for a sample patient in one slice. **a** Dose distribution for 3D-CRT AP–PA technique. **b** Dose distribution for 3D-CRT with 8 coplanar fields technique. **c** Dose distribution for IMRT technique. **d** Dose distribution for VMAT technique

**Fig. 3:**
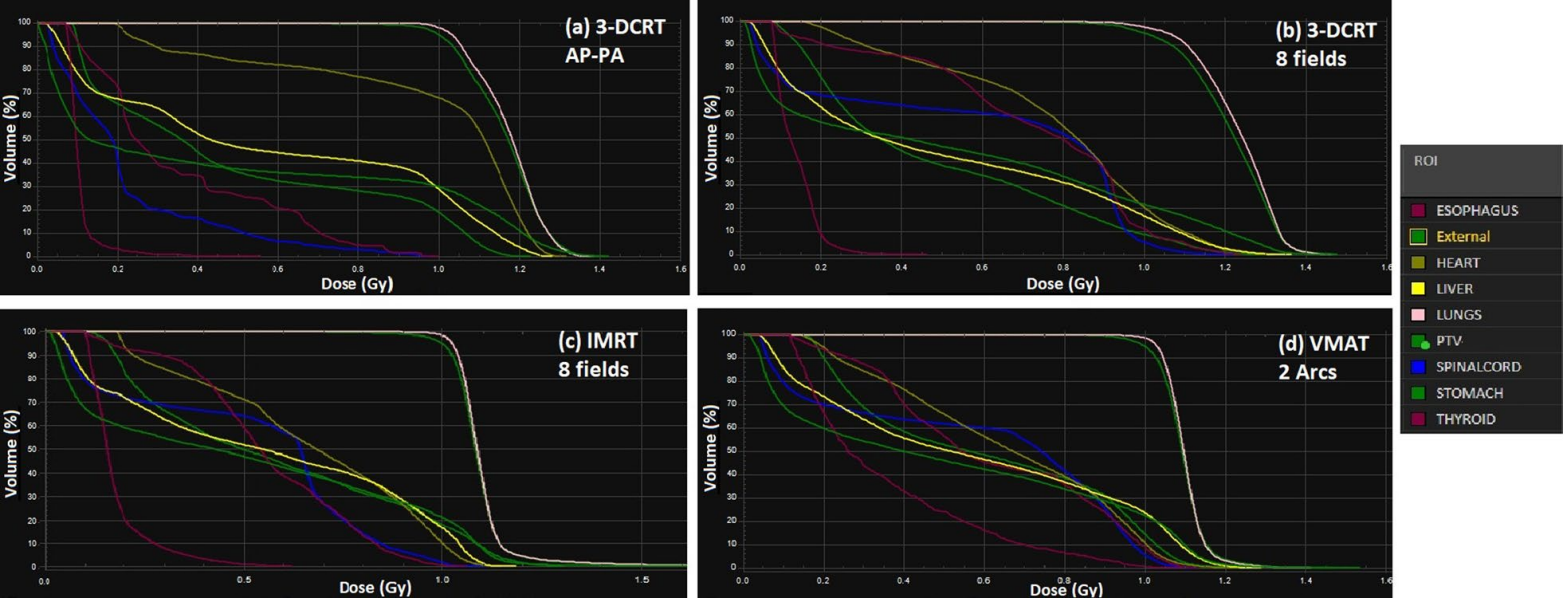
Dose volume histograms (DVHs) of whole lung LDRT with different delivery techniques and 1 Gy prescription for a sample patient in one slice. **a** DVH of 3D-CRT AP–PA technique. **b** DVH of 3D-CRT with 8 coplanar fields technique. **c** DVH of IMRT technique. **d** DVH of VMAT technique

Figure 2 presented the dose distribution of whole lung LDRT with different delivery techniques and 1 Gy prescription for a patient in one slice. Figure 3 also illustrated the dose volume histograms (DVHs) of these techniques. It can be seen that at least 95% of lungs volume receive the 95% of the prescribed dose homogeneously in all techniques. However, the heart and chest wall (breast in females) receive higher doses (close to lung prescribed dose) in the AP–PA technique (Figs. 2a, 3a). In intensity modulated techniques, the heart and chest wall receive lower doses (Fig. 2c, d) due to modulation of irradiation for sparing these structures. Furthermore, it seems that the eight fields 3D-conformal technique showed better sparing of heart and chest wall (or breast) compared to the AP–PA technique (Figs. 2b, 3b).

The mean ± standard deviation values of organ doses (mean and maximum dose), HI, and CI for different radiotherapy techniques were presented in Table 2. These doses were used for calculating the radiation induced cancer risks. In the 3D-CRT AP–PA technique, the highest radiation doses were observed in the lung, breast (for women), and heart, in the prescribed dose range. However, in IMRT and VMAT delivery techniques, the heart and breast doses and most of the healthy organ doses (except the thyroid dose) were lower than the 3D-CRT AP–PA technique. Regarding the statistical analysis, heart (mean and max), breast (mean and max), and stomach (mean) doses and also maximum dose in the body were significantly lower in IMRT and VMAT compared to 3D-CRT techniques (*P* < 0.04). For example, mean doses of heart and breast (for women) were 31% and 54% lower in IMRT and VMAT techniques. However, there were not any significant differences between IMRT and VMAT techniques regarding the organ doses. The 3D-CRT with eight coplanar fields showed lower heart and breast mean doses than the AP–PA technique, with no statistical differences with intensity modulated techniques (*P* < 0.05). Furthermore, the organ dose differences between males and females were minor (*P* > 0.2), except for the breast in all the assessed treatment delivery techniques.

**Table 2:** Calculated mean ± standard deviation values of organ doses for different radiotherapy techniques

Dosimetric parameters	3D-CRT AP–PA	3D-CRT 8 fields	IMRT	VMAT
PTV CI	0.94 ± 0.06	0.94 ± 0.04	0.97 ± 0.05	0.95 ± 0.05
PTV HI	0.38 ± 0.07	0.37 ± 0.07	0.30 ± 0.06	0.27 ± 0.04
Lung mean dose (Gy)	1.17 ± 0.15	1.23 ± 0.18	1.09 ± 0.11	1.1 ± 0.13
Lungs max dose (Gy)	1.33 ± 0.19	1.40 ± 0.22	1.43 ± 0.15	1.27 ± 0.17
Heart mean dose (Gy)	0.96 ± 0.10	0.77 ± 0.09	0.66 ± 0.05	0.65 ± 0.07
Heart max dose (Gy)	1.27 ± 0.12	1.25 ± 0.11	1.08 ± 0.08	1.12 ± 0.08
Breast mean dose (Gy)	1.22 ± 0.15	0.64 ± 0.05	0.55 ± 0.04	0.59 ± 0.05
Breast max dose (Gy)	1.35 ± 0.17	0.95 ± 0.07	0.72 ± 0.06	0.77 ± 0.06
Liver mean dose (Gy)	0.57 ± 0.06	0.49 ± 0.05	0.55 ± 0.06	0.57 ± 0.07
Liver max dose (Gy)	1.24 ± 0.11	1.25 ± 0.14	1.1 ± 0.09	1.19 ± 0.1
Stomach mean dose (Gy)	0.58 ± 0.04	0.54 ± 0.05	0.43 ± 0.05	0.45 ± 0.05
Stomach max dose (Gy)	1.15 ± 0.11	1.2 ± 0.10	0.98 ± 0.08	1.13 ± 0.08
Thyroid mean dose (Gy)	0.11 ± 0.01	0.14 ± 0.02	0.18 ± 0.03	0.35 ± 0.03
Thyroid max dose (Gy)	0.28 ± 0.03	0.29 ± 0.04	0.49 ± 0.05	0.98 ± 0.08
Esophagus mean dose (Gy)	0.35 ± 0.03	0.71 ± 0.06	0.56 ± 0.06	0.61 ± 0.07
Esophagus max dose (Gy)	0.95 ± 0.08	1.19 ± 0.09	0.98 ± 0.07	1.08 ± 0.08
Spinal cord mean dose (Gy)	0.22 ± 0.02	0.59 ± 0.05	0.51 ± 0.04	0.58 ± 0.05
Spinal cord maximum dose (Gy)	0.92 ± 0.10	1.1 ± 0.10	1.01 ± 0.09	1.06 ± 0.08
Maximum dose in body (Gy)	1.35 ± 0.11	1.36 ± 0.12	1.21 ± 0.11	1.23 ± 0.09
Integral dose (Gy.cc)	12,581.1 ± 1042.2	11,594.3 ± 1015.5	12,334.4 ± 1150.1	12,446.6 ± 1072.9

The calculated CI indexes were similar in all the techniques. However, the HI indexes were lower (i.e. better) in intensity modulated techniques (*P* < 0.03) with no significant differences between IMRT and VMAT treatment plans (*P* > 0.6).

The Integral dose among the assessed techniques had not significant difference (*P* > 0.08), indicating the radiation induced cancer incidence risks due to whole lung low dose radiotherapy (with a prescribed dose equal to 1 Gy) in different techniques for a 30 years man and woman were presented in Table 3. The radiation induced cancer mortality risks were also presented in Table 4.

**Table 3:** Radiation induced cancer incidence risks (mean ± standard deviation) due to whole lung low dose radiotherapy (with a prescribed dose equal to 1 Gy) in different techniques for a 30 years old man and woman

Cancer site	3D-CRT AP–PA	3D-CRT 8 fields	IMRT	VMAT
*Male*				
Stomach	0.162 ± 0.011	0.151 ± 0.014	0.120 ± 0.014	0.126 ± 0.014
Liver	0.125 ± 0.013	0.108 ± 0.011	0.121 ± 0.013	0.125 ± 0.015
Lung	1.185 ± 0.158	1.292 ± 0.189	1.144 ± 0.116	1.155 ± 0.137
Other	0.416 ± 0.040	0.356 ± 0.038	0.277 ± 0.032	0.307 ± 0.038
Thyroid	0.010 ± 0.001	0.013 ± 0.002	0.016 ± 0.003	0.031 ± 0.003
All solids	1.942 ± 0.220	1.920 ± 0.210	1.679 ± 0.196	1.745 ± 0.202
*Female*				
Stomach	0.173 ± 0.014	0.194 ± 0.018	0.155 ± 0.018	0.162 ± 0.018
Liver	0.057 ± 0.006	0.049 ± 0.005	0.055 ± 0.006	0.057 ± 0.007
Lung	2.831 ± 0.363	2.977 ± 0.436	2.638 ± 0.266	2.662 ± 0.315
Breast	3.087 ± 0.430	2.226 ± 0.127	2.226 ± 0.101	1.948 ± 0.126
Other	0.435 ± 0.041	0.373 ± 0.039	0.290 ± 0.033	0.321 ± 0.035
Thyroid	0.044 ± 0.004	0.057 ± 0.008	0.074 ± 0.012	0.143 ± 0.012
All solids	6.664 ± 0.548	5.269 ± 0.490	4.603 ± 0.462	4.838 ± 0.492

Risk values are presented as incidence probability per 100 individuals

**Table 4:** Radiation induced cancer mortality risks (mean ± standard deviation) due to whole lung low dose radiotherapy (with a prescribed dose equal to 1 Gy) in different techniques for a 30 years old man and woman

Cancer site	3D-CRT AP–PA	3D-CRT 8 fields	IMRT	VMAT
*Male*				
Stomach	0.093 ± 0.006	0.086 ± 0.008	0.069 ± 0.008	0.072 ± 0.008
Liver	0.091 ± 0.011	0.078 ± 0.008	0.088 ± 0.010	0.091 ± 0.011
Lung	1.252 ± 0.161	1.316 ± 0.193	1.166 ± 0.118	1.177 ± 0.139
Other	0.197 ± 0.019	0.169 ± 0.018	0.132 ± 0.015	0.146 ± 0.018
All solids	1.633 ± 0.123	1.650 ± 0.121	1.455 ± 0.099	1.486 ± 0.103
*Female*				
Stomach	0.122 ± 0.008	0.113 ± 0.011	0.090 ± 0.011	0.094 ± 0.011
Liver	0.051 ± 0.005	0.044 ± 0.005	0.049 ± 0.005	0.051 ± 0.006
Lung	2.492 ± 0.322	2.620 ± 0.383	2.322 ± 0.234	2.343 ± 0.277
Breast	0.744 ± 0.104	0.390 ± 0.031	0.336 ± 0.024	0.360± 0.031
Other	0.216 ± 0.021	0.185 ± 0.020	0.144 ± 0.016	0.159 ± 0.018
All solids	3.626 ± 0.295	3.353 ± 0.293	3.941 ± 0.313	3.008 ± 0.283

Risk values are presented as incidence probability per 100 individuals

The radiation induced cancer and mortality risks for adult patients of different ages (20–80 years) and different radiotherapy techniques were presented in the “Appendix” as tables.

Lifetime lung cancer incident risks for all the delivery techniques were statistically similar (*P* > 0.4). Cancer risks for organs located closer to the lung like breast and stomach were significantly higher in 3D-CRT techniques than IMRT or VMAT techniques (*P* < 0.03). However, eight fields 3D-conformal had significantly lower breast cancer risk compared to the 3D-CRT AP–PA technique (*P* < 0.01). On the other hand, the thyroid had lower doses and lower cancer risks in 3D-conformal techniques (*P* < 0.01). Furthermore, the 3D-CRT technique with AP–PA fields had the lowest mean and maximum dose for thyroid and esophagus because the primary radiations from anterior and posterior fields did not pass these structures even partially.

Radiotherapy with higher prescribed doses resulted in higher cancer risks in all the delivery techniques, because the BEIR VII-phase 2 preferred model considered a linear relationship between the organs doses and cancer risks in low doses up to 2 Gy [22].

**Fig. 4:**
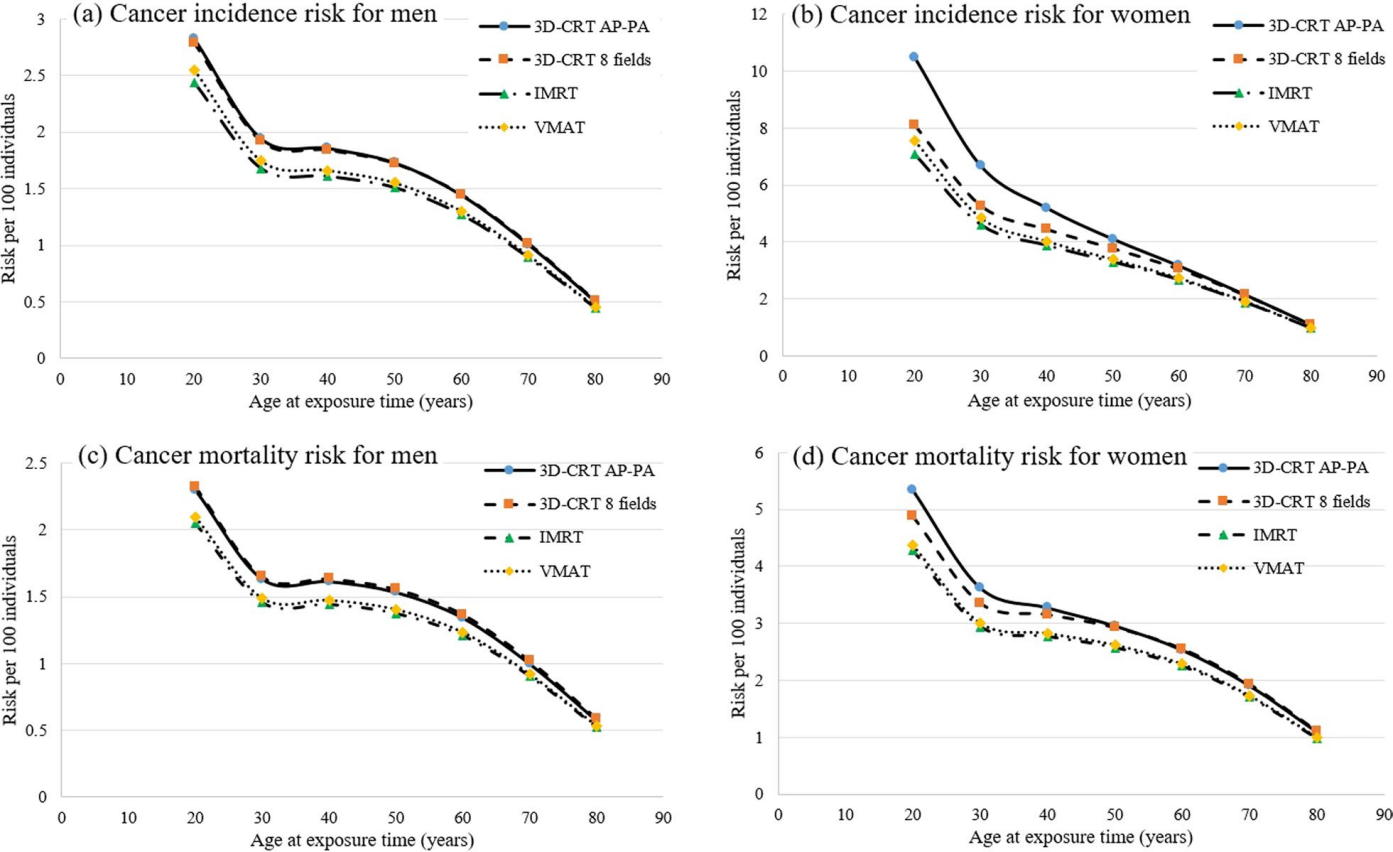
The radiation induced lifetime solid tumors cancer incidence and mortality risks for patients undergoing whole lung LDRT with different radiotherapy techniques at different ages. **a** Cancer incidence risks for male patients at different ages. **b** Cancer incidence risks for female patients at different ages. **c** Cancer mortality risks for male patients at different ages. **d** Cancer mortality risks for female patients at different ages

The radiation induced  lifetime solid tumors cancer incidence and mortality risks at various ages of exposure for were illustrated in Fig. 4 for different delivery techniques. As can be noticed, the lifetime attributed risk had higher values and also higher differences at lower ages among different techniques. The cancer risk differences among different delivery techniques were reduced with increasing the age of exposure. This figure shows that the total risks were higher for females due to the higher probability of cancer incidence for women in cancer estimation models. Furthermore, the breasts, as the women’s high radiosensitive organs, are located near the lungs and were irradiated fully or partially in different delivery techniques.

Another side effect of low dose radiations, the excessive relative risk of heart coronary diseases due to LDRT irradiations showed significant differences between the radiation delivery techniques (*P* < 0.045). The ERR values for 3D-CRT AP–PA, eight fields 3D-CRT, IMRT, and VMAT techniques were 7.10 ± 0.74%, 5.70 ± 0.67%, 4.88 ± 0.37%, and 4.81 ± 0.52%, respectively. ERR values obtained from the IMRT and VMAT techniques did not show significant differences and were lower than 3D-CRT techniques.

## Discussion

We evaluated the cancer risks induced by whole lung LDRT of COVID-19 pneumonia in different techniques as the main side effect of low dose radiotherapy. There are other parameters used in evaluating the radiotherapy side effects like effective dose, biologically effective dose, and organ complications. In low dose radiotherapy (total dose < 1.5 Gy), there was no report of complications for the structures located in the chest region except the heart. Although, the effective dose could compare the radiation effects of different radiotherapy techniques. However, it was shown that this parameter could not appropriately predict the radiobiological outcomes for low-dose radiotherapy. For example, while male and female adults had received almost the same effective dose, radiation induced cancer risks are entirely different. Furthermore, the age of exposure as an important factor can not be considered in the effective dose. Thus, the effective dose is not recommended for epidemiological evaluations [34], and in this study, we estimated the excess radiation induced cancer risks as the main side effects of whole lung LDRT.

It was proposed that LDRT may induce anti-inflammatory effects and helps to reduce or prevent the cytokine storm [6, 20]. Historical reports suggest radiation doses between 0.35 and 0.5 Gy for lung LDRT as an optimal dose, and higher doses may induce excessive inflammation in pneumonia patients [35]. Recent studies and clinical trials showed that higher doses up to 1.5 Gy were effective for treating the COVID-19 pneumonia [5, 18]. Although we found that the IMRT and VMAT delivery techniques lead to significantly lower radiation induced cancer risks compared to AP–PA conformal technique, but we think that the effectiveness of different LDRT dose prescriptions must be compared in a clinical trial study to find the optimum LDRT dose prescription protocol for treating COVID-19 pneumonia. Therefore, we just presented the excess cancer risks results of LDRT with 1 Gy X-ray prescribed dose for different radiation delivery techniques. Since the cancer induced risk probability relation with absorbed dose is linear in low doses, one can easily calculate the cancer risks at different LDRT doses by multiplying the estimated cancer risks in this study by the radiation dose (at Gy).

Although there are several clinical trials about the treatment effectiveness of LDRT on COVID-19 pneumonia with different dose prescriptions, there is a frustrating lack of detail about technical delivery techniques. We tried to use different delivery techniques and evaluate their risks to prepare appropriate data for clinicians about choosing the delivery techniques in different situations. A simple AP/PA treatment method provided relatively similar conformity indexes in comparison with more advanced techniques (like IMRT) and may be appropriate for initial clinical trials. But we think that other delivery techniques like 3D-conformal with the higher number of fields, IMRT, and VMAT are better choices for clinical applications due to their significantly lower cancer risks, especially for younger patients. Our results showed that IMRT and VMAT had lower cancer risks compared to 3D-conformal techniques. The superiority of intensity modulated techniques may be related to their modulated radiation which could better spare the organs at risk like the breast, heart, and liver. Furthermore, these techniques could be assessed in future clinical trial designs.

Several studies [33–36] mentioned that intensity modulated techniques had higher low dose regions in radiotherapy. Therefore, secondary cancer probabilities are higher for these techniques, although the risk of organ malfunction/dysfunction and organ doses (mean and maximum doses) are lower. This may be a little confusing regarding our results. It must be mentioned that the total dose of one radiotherapy course is much higher than the LDRT. For example, the radiation dose for treatment of locally advanced prostate cancer is usually higher than 70 Gy in 35 fractions, and the radiation doses for treating non-small cell lung cancers at stage I-III are generally higher than 50 Gy in equally 2 Gy per fraction regime. Therefore, the low dose regions (with lower than 10% of prescription dose) have doses as high as 5 Gy, much higher than lung LDRT.

Furthermore, organ complications like dysfunction may occur in high dose regions, and secondary cancer risks are much possible where the organ complications have lower probabilities. LDRT dose levels are remarkably lower than the radiotherapy regimens (30–100 times lower). There are no radiation effects for lung at less than 7 Gy X-ray doses, and lung LDRT doses (0.5–1.5 Gy) are well below this threshold [37, 38]. In this situation, the cancer risk is directly correlated with dose, as we showed in the results of this study.

In radiotherapy, estimation of radiation induced cancer risks can be divided into two dose regions named medium dose (region positioned between 5 and 50% isodose lines) and low dose regions (regions lower than the 5% of the prescription dose) [36]. Radiobiological factors at the cellular level including DNA mutations, cell survival, cell repair and repopulation which occur during fractionated exposures must be considered in a comprehensive model which incorporates the induction of cancers in medium dose regions. Several models are addressing these issues in the literature [37–40]. However, it was shown that at low doses, different fractionation regimes have the same biological results [38]. Schneider et al. [37] proposed a model for medium to small doses, the linear no-threshold model, based on the information recorded from atomic bomb survivors. This model was described in details by Sánchez-Nieto et al. [36]. The results of the previous studies indicate that the linear models used in radiation protection which is explained by the ICRP [25] would be suitable for cancer estimation in low dose regions (< 4 Gy). We used the linear model from the BEIR-VII report [22] instead of the ICRP model [34]. The ICRP model did not consider sex and age specificities in the risk estimations. Furthermore, cancer risks for different sites and organs are available in the BEIR-VII model. The Schneider model [23, 24, 37] considers the dose distributions in the radiosensitive organs for calculating the cancer risks. However, the main focus of the Schneider model is on the doses above 1 Gy in radiotherapy, and we think that this model is more suitable for cancer estimation in medium dose regions. We also must mention that this work's aim is not to decide which model is the most appropriate. It is possible to use other models for cancer estimation like the ICRP or Schneider model for cancer estimation of lung LDRT in COVID-19 pneumonia patients.

It was reported that the uncertainties associated with each of the risk models developed for cancer incidence and mortality estimation, including ICRP [25], BEIR [22], and UNSCEAR [31] are in the range of the variation between the models [41]. We have chosen the BEIR VII model as it provides the parameters for specific organs for each sex and includes a parameter describing incidence with age at exposure and attained age.

There are significant uncertainties in estimated LAR values presented in the BEIR VII report [22]. The report gives an estimate of LAR for solid cancer incidence in the female breast as 310 cases (95% confidence intervals (CI) 160, 610) per 100 000 individuals exposed to 0.1 Gy. For lung cancer incidence in females of the same population and dose, LAR is given as 300 (95% CI 120, 780). Uncertainties for other sites have similar levels. Although there are remarkable uncertainties in cancer risk estimation using the BEIR-VII model due to the lack of epidemiological information, other cancer risk estimation models suffer from similar uncertainties. Harrison et al. [42] reported their reluctance to estimate cancer risks based on the measured organ doses. However, other groups have found the risk models useful to compare different treatment methods while accepting the large uncertainties in an absolute risk values [22, 43].

As a comparison between the induced cancer risks of COVID-19 LDRT and other radiological modalities, it must be mentioned that LDRT with the dose of 0.5–1.5 Gy delivers much higher ionizing radiations to patients in comparison with diagnostic radiological examinations like computed tomography or chest radiographies. For example, lung dose of 50 cGy, corresponds to approximately 11 thorax CTs, 8 CT scans of abdomen with the highest dose protocols, 7 cardiac CTs, 7 4DCT, and 7 whole body high-quality PET/CTs. If higher dose prescriptions are administrated for COVID-19 pneumonia treatment, the equivalent examinations should be increased proportionally. Furthermore, it must be mentioned that competing mortality risk factors in patients with COVID-19 pneumonia is likely to be substantially higher than the general population, therefore radiation induced cancer risks will be small compared to other factors.

Linear dose–response relationships between the risk of coronary heart diseases after radiotherapy and radiation dose were reported [26, 32]. Darby et al. [32] reported an ERR of 7.4%/Gy; however, Nimwegen et al. [26] found an ERR of 4.2%/Gy for radiation induced coronary heart diseases after radiotherapy of breast cancers and Hodgkin lymphoma, respectively. The confidence intervals of the ERRs in both studies overlap, it is likely that uncertainties in both data sets partially explain the difference in the magnitude of the ERRs. Most of the previous studies [32, 44] reported that there is no threshold dose between mean heart dose and coronary heart diseases. Although a previous study [45] only showed increased risks of the diseases after a mean dose exceeding 15 Gy. In a more recent study on the data of 24,214 survivors from childhood cancer from 1970 to 1999, it was reported that late cardiac disease risk increases with Dmean ≥ 10 Gy, V20 ≥ 0.1%, and V5 ≥ 50%, and risks were significantly higher than previously estimated for survivors with Dmean in the range of 20–29.9 Gy. Although they did not present the conclusion of cardiac disease risks with low Dmean values (<1Gy), higher risks can be expected in low dosess compared to older studies [46].

The dosimetric parameter used for assessing the heart dose in both of the previous studies was the mean heart dose. Although the doses to the coronary arteries were not estimated; however, Darby et al. [32] estimated the radiation dose to the left anterior descending coronary artery but found that the mean heart dose was a better predictor of the rate of major coronary events than the mean dose to the left anterior descending artery, as the dose to the coronary arteries was an uncertain measure. In conclusion, the mean radiation dose to the heart is an important risk factor for the development of coronary heart diseases.

The dose calculation algorithm used in this study was Collapsed Cone Convolution Superposition algorithm (CCCS). The gold standard dose calculation method for dosimetry ionizing radiation is Monte Carlo. However, for large field sizes and mega voltage X-ray energies, discrepancies in dose calculation are lower than 4% in inhomogeneity boundaries and lower than 1% in other regions for CCCS algorithms [47]. The CCCS algorithm has a higher agreement with Monte Carlo in inhomogeneous regions compared to other commercial radiotherapy dosimetry algorithms [48]. BEIR VII model did not provided LAR values for estimating some of the cancers like the radiation induced excess oesophageal cancer risks which is the third most common supradiaphragmatic cancer. This could be assumed as one of the important limitations of our study. Althogh it provided the LAR values for radiation induced risks of all solid cancers.

## Conclusion

Regarding our results, all the radiotherapy techniques had low cancer risks, and a simple AP/PA treatment method provided relatively similar homogeneity and conformity indexes in comparison with more advanced techniques (like IMRT) and may be appropriate for initial clinical trials. The overall risks induced by IMRT and VMAT radiotherapy techniques were lower than the 3D-CRT techniques and can be used clinically in younger patients or patients having greater concerns about future cancers. Higher cancer risks except the lungs are related to breast, and stomach which must be considered for lung LDRT. Furthermore, intensity modulated methods or 3D-CRT method with a high number of fields could be an appropriate radiotherapy technique for women in which the cancer risks are higher due to irradiation of breast tissue, especially in the centers having lower setup errors and radiation uncertainties.

## Data Availability

All data obtained during the current study are available from the corresponding author on reasonable request.

## Appendix

The Radiation induced cancer and mortality risks for adult patients in different ages (20–80 years) and different radiotherapy techniques were presented in the following tables (Tables 5, 6, 7, 8, 9, 10, 11 and 12).

Tables 5 and 6 represent the radiation induced cancer and mortality risks respectively, for 3D-CRT AP–PA technique.

**Table 5:** The mean lifetime attributed cancer induction risks due to whole lung low dose radiotherapy (with a prescribed dose equal to 1 Gy) in 3D-CRT AP–PA technique for adult male and female patients at different ages

	Age at exposure time (years)						
	20	30	40	50	60	70	80
*Male*							
Stomach	0.232	0.162	0.157	0.145	0.116	0.081	0.041
Liver	0.171	0.125	0.120	0.108	0.080	0.046	0.017
Lung	1.743	1.785	1.217	1.182	1.041	0.761	0.398
Other	0.655	0.416	0.361	0.294	0.206	0.120	0.048
Thyroid	0.023	0.010	0.003	0.001	0.000	0.000	0.000
All solids	2.825	1.941	1.858	1.730	1.443	1.007	0.504
*Female*							
Stomach	0.250	0.173	0.168	0.154	0.130	0.091	0.053
Liver	0.080	0.057	0.057	0.051	0.040	0.029	0.011
Lung	4.048	2.831	2.808	2.691	2.352	1.720	0.901
Breast	5.234	3.087	1.720	0.854	0.378	0.146	0.049
Other	0.678	0.435	0.380	0.311	0.229	0.143	0.063
Thyroid	0.124	0.045	0.015	0.004	0.001	0.000	0.000
All solids	10.466	6.664	5.184	4.097	3.156	2.148	1.088

Risk values are presented as incidence probability per 100 individuals

**Table 6:** The mean lifetime attributed cancer mortality risks due to whole lung low dose radiotherapy (with a prescribed dose equal to 1 Gy) in 3D-CRT AP–PA technique for adult male and female patients at different ages

	Age at exposure time (years)						
	20	30	40	50	60	70	80
*Male*							
Stomach	0.122	0.093	0.087	0.075	0.064	0.046	0.023
Liver	0.131	0.091	0.091	0.080	0.068	0.046	0.023
Lung	1.767	1.819	1.252	1.217	1.088	0.831	0.491
Other	0.281	0.197	0.185	0.162	0.122	0.076	0.036
All solids	2.301	1.633	1.615	1.534	1.342	0.998	0.573
*Female*							
Stomach	0.168	0.122	0.116	0.110	0.093	0.075	0.046
Liver	0.068	0.051	0.046	0.046	0.040	0.029	0.017
Lung	3.569	2.492	2.480	2.387	2.141	1.638	0.948
Breast	1.232	0.744	0.427	0.232	0.110	0.061	0.024
Other	0.309	0.216	0.204	0.181	0.145	0.099	0.050
All solids	5.209	3.597	3.334	3.041	2.594	1.942	1.114

Risk values are presented as incidence probability per 100 individuals

Tables 7 and 8 represent the radiation induced cancer and mortality risks respectively, for 3D-CRT 8 fields technique.

**Table 7:** The mean lifetime attributed cancer induction risks due to whole lung low dose radiotherapy (with a prescribed dose equal to 1 Gy) in 3D-CRT 8 fields technique for adult male and female patients at different ages

	Age at exposure time (years)						
	20	30	40	50	60	70	80
*Male*							
Stomach	0.216	0.151	0.146	0.135	0.108	0.076	0.038
Liver	0.147	0.108	0.103	0.093	0.069	0.039	0.015
Lung	1.833	1.292	1.279	1.242	1.095	0.800	0.418
Other	0.562	0.356	0.310	0.252	0.176	0.103	0.041
Thyroid	0.029	0.013	0.004	0.001	0.000	0.000	0.000
All solids	2.787	1.920	1.842	1.724	1.448	1.017	0.512
*Female*							
Stomach	0.281	0.194	0.189	0.173	0.146	0.103	0.059
Liver	0.069	0.049	0.049	0.044	0.034	0.025	0.010
Lung	4.256	2.977	2.952	2.829	2.472	1.808	0.947
Breast	2.746	1.619	0.902	0.488	0.198	0.077	0.026
Other	0.581	0.373	0.326	0.266	0.196	0.122	0.054
Thyroid	0.158	0.057	0.020	0.006	0.001	0.000	0.000
All solids	8.090	5.269	4.438	3.766	3.048	2.135	1.096

Risk values are presented as incidence probability per 100 individuals

**Table 8:** The mean lifetime attributed cancer mortality risks due to whole lung low dose radiotherapy (with a prescribed dose equal to 1 Gy) in 3D-CRT 8 fields technique for adult male and female patients at different ages

	Age at exposure time (years)						
	20	30	40	50	60	70	80
*Male*							
Stomach	0.113	0.086	0.081	0.070	0.059	0.043	0.022
Liver	0.113	0.078	0.078	0.069	0.059	0.039	0.020
Lung	1.857	1.316	1.316	1.279	1.144	0.873	0.517
Other	0.241	0.169	0.158	0.139	0.104	0.065	0.031
All solids	2.325	1.650	1.634	1.557	1.367	1.021	0.588
*Female*							
Stomach	0.157	0.113	0.108	0.103	0.086	0.070	0.043
Liver	0.059	0.044	0.039	0.039	0.034	0.025	0.015
Lung	3.752	2.620	2.608	2.509	2.251	1.722	0.996
Breast	0.646	0.390	0.224	0.122	0.058	0.032	0.013
Other	0.265	0.185	0.175	0.155	0.124	0.085	0.043
All solids	4.878	3.353	3.153	2.927	2.553	1.933	1.110

Risk values are presented as incidence probability per 100 individuals

Tables 9 and 10 represent the radiation induced cancer and mortality risks respectively, for IMRT technique.

**Table 9:** The mean lifetime attributed cancer induction risks due to whole lung low dose radiotherapy (with a prescribed dose equal to 1 Gy) in IMRT technique for adult male and female patients at different ages

	Age at exposure time (years)						
	20	30	40	50	60	70	80
*Male*							
Stomach	0.172	0.120	0.116	0.108	0.086	0.060	0.030
Liver	0.165	0.121	0.116	0.105	0.077	0.044	0.017
Lung	1.624	1.145	1.134	1.101	0.970	0.709	0.371
Other	0.437	0.277	0.241	0.196	0.137	0.080	0.032
Thyroid	0.038	0.016	0.005	0.002	0.001	0.000	0.000
All solids	2.436	1.679	1.611	1.511	1.271	0.893	0.449
*Female*							
Stomach	0.224	0.155	0.151	0.138	0.116	0.082	0.047
Liver	0.077	0.055	0.055	0.050	0.039	0.028	0.011
Lung	3.771	2.638	2.616	2.507	2.191	1.602	0.839
Breast	2.360	1.392	0.776	0.385	0.171	0.066	0.022
Other	0.452	0.290	0.253	0.207	0.153	0.095	0.042
Thyroid	0.203	0.074	0.025	0.007	0.002	0.001	0.000
All solids	7.087	4.603	3.876	3.294	2.670	1.873	0.962

Risk values are presented as incidence probability per 100 individuals

**Table 10:** The mean lifetime attributed cancer mortality risks due to whole lung low dose radiotherapy (with a prescribed dose equal to 1 Gy) in IMRT technique for adult male and female patients at different ages

	Age at exposure time (years)						
	20	30	40	50	60	70	80
*Male*							
Stomach	0.090	0.069	0.065	0.056	0.047	0.034	0.017
Liver	0.127	0.088	0.088	0.077	0.066	0.044	0.022
Lung	1.646	1.166	1.166	1.134	1.014	0.774	0.458
Other	0.188	0.132	0.123	0.108	0.081	0.050	0.024
All solids	2.050	1.455	1.442	1.374	1.208	0.903	0.521
*Female*							
Stomach	0.125	0.090	0.086	0.082	0.069	0.056	0.034
Liver	0.066	0.050	0.044	0.044	0.039	0.028	0.017
Lung	3.325	2.322	2.311	2.224	1.995	1.526	0.883
Breast	0.556	0.336	0.193	0.105	0.050	0.028	0.011
Other	0.206	0.144	0.136	0.120	0.097	0.066	0.034
All solids	4.250	2.935	2.720	2.481	2.116	1.584	0.909

Risk values are presented as incidence probability per 100 individuals

Tables 11 and 12 represent the radiation induced cancer and mortality risks respectively, for VMAT technique.

**Table 11:** The mean lifetime attributed cancer induction risks due to whole lung low dose radiotherapy (with a prescribed dose equal to 1 Gy) in VMAT technique for adult male and female patients at different ages

	Age at exposure time (years)						
	20	30	40	50	60	70	80
*Male*							
Stomach	0.180	0.126	0.122	0.113	0.090	0.063	0.032
Liver	0.171	0.125	0.120	0.108	0.080	0.046	0.017
Lung	1.639	1.155	1.144	1.111	0.979	0.715	0.374
Other	0.484	0.307	0.267	0.217	0.152	0.088	0.036
Thyroid	0.074	0.032	0.011	0.004	0.001	0.000	0.000
All solids	2.547	1.745	1.662	1.552	1.302	0.912	0.458
*Female*							
Stomach	0.234	0.162	0.158	0.144	0.122	0.086	0.050
Liver	0.080	0.057	0.057	0.051	0.040	0.029	0.011
Lung	3.806	2.662	2.640	2.530	2.211	1.617	0.847
Breast	2.531	1.493	0.832	0.413	0.183	0.071	0.024
Other	0.501	0.321	0.281	0.229	0.169	0.105	0.047
Thyroid	0.396	0.144	0.049	0.014	0.004	0.001	0.000
All solids	7.547	4.838	4.016	3.382	2.728	1.908	0.978

Risk values are presented as incidence probability per 100 individuals

**Table 12:** The mean lifetime attributed cancer mortality risks due to whole lung low dose radiotherapy (with a prescribed dose equal to 1 Gy) in VMAT technique for adult male and female patients at different ages

	Age at exposure time (years)						
	20	30	40	50	60	70	80
*Male*							
Stomach	0.095	0.072	0.068	0.059	0.050	0.036	0.018
Liver	0.131	0.091	0.091	0.080	0.068	0.046	0.023
Lung	1.661	1.177	1.177	1.144	1.023	0.781	0.462
Other	0.208	0.146	0.136	0.119	0.090	0.056	0.026
All solids	2.094	1.486	1.472	1.402	1.231	0.918	0.529
*Female*							
Stomach	0.131	0.095	0.090	0.086	0.072	0.059	0.036
Liver	0.068	0.051	0.046	0.046	0.040	0.029	0.017
Lung	3.355	2.343	2.332	2.244	2.013	1.540	0.891
Breast	0.596	0.360	0.207	0.112	0.053	0.030	0.012
Other	0.228	0.160	0.150	0.133	0.107	0.073	0.037
All solids	4.378	3.008	2.824	2.621	2.285	1.729	0.993

Risk values are presented as incidence probability per 100 individuals

## Abbreviations

3D-CRT: 3-Dimentional-conformal radiotherapy
AP–PA: Anterior posterior–posterior anterior
IMRT: Intensity modulated radiation therapy
VMAT: Volumetric modulated arc therapy
LDRT: Low dose radiotherapy
ARDS: Acute respiratory distress syndrome
BEIR: Biologic effects of ionizing radiation
HU: Hounsfield unit
PTV: Planning tumor volume
HI: Homogeneity index
CI: Conformity index
